# Association of *Helicobacter pylori* infection with the risk of metabolic syndrome and insulin resistance: an updated systematic review and meta-analysis

**DOI:** 10.1186/s13098-021-00765-x

**Published:** 2021-12-18

**Authors:** Mobin Azami, Hamid Reza Baradaran, Hojat Dehghanbanadaki, Parisa Kohnepoushi, Lotfolah Saed, Asra Moradkhani, Farhad Moradpour, Yousef Moradi

**Affiliations:** 1 Student Research Committee, Kurdistan University of Medical Sciences, Sanandaj, Iran; 2 Ageing Clinical & Experimental Research Team, Institute of Applied Health Sciences, University of Aberdeen, Aberdeen, UK; 3 Endocrine Research Center, Institute of Endocrinology and Metabolism, Iran University of Medical Sciences, Sanandaj, Iran; 4 Students Scientific Research Center, Tehran University of Medical Sciences, Tehran, Iran; 5 Department of Endocrinology, Faculty of Medicine, Kurdistan University of Medical Science, Sanandaj, Iran; 6 Social Determinants of Health Research Center, Research Institute for Health Development, Kurdistan University of Medical Science, Sanandaj, Iran; 7 Department of Biostatics and Epidemiology, Faculty of Medicine, Kurdistan University of Medical Science, Sanandaj, Iran

**Keywords:** *Helicobacter pylori*, Insulin resistance, Metabolic syndrome, Systematic review, Meta-analysis

## Abstract

**Background:**

Conflicting results of recent studies on the association between Helicobacter pylori (*H. pylori*) infection and the risk of insulin resistance and metabolic syndrome explored the need for updated meta-analysis on this issue. Therefore, this systematic review aimed to estimate the pooled effect of *H. pylori* infection on the risk of insulin resistance and metabolic syndrome.

**Methods:**

To identify case–control studies and cohort studies evaluating the association of *H. pylori* infection with insulin resistance and metabolic syndrome, a comprehensive literature search was performed from international databases including Medline (PubMed), Web of Sciences, Scopus, EMBASE, and CINHAL from January 1990 until January 2021. We used odds ratio with its 95% confidence interval to quantify the effect of case–control studies and risk ratio with its 95% CI for the effect of cohort studies.

**Results:**

22 studies with 206,911 participants were included for meta-analysis. The pooled estimate of odds ratio between *H. pylori* infection and metabolic syndrome in case–control studies was 1.19 (95% CI 1.05–1.35; I^2^ = 0%), and in cohort studies, the pooled risk ratio was 1.31 (95% CI 1.13–1.51; I^2^ = 0%). Besides, case–control studies showed the pooled odds ratio of 1.54 (95% CI 1.19–1.98; I^2^ = 6.88%) for the association between *H. pylori* infection and insulin resistance.

**Conclusion:**

In this meta-analysis, the results showed that there was a possibility of metabolic syndrome and insulin resistance in case of *H. pylori* infection.

## Introduction

*Helicobacter pylori* (*H. pylori*) is a gram-negative bacterium and a very common pathogen, which has infected more than half of the world's population. To diagnose this infection, both invasive tests such as upper gastrointestinal endoscopy with gastric biopsy and non-invasive tests such as the urea respiration test, stool test, and blood test are available in clinical practice [1–3]. Although the incidence of *H. pylori* infection is declining worldwide, the infection is still a communicable disease with serious health consequences. There are many differences in the distribution of *H. pylori* infection in the population between developed and developing countries. In this instance, almost 80% of infected population in developing countries such as India and Vietnam are before the age of 20 while in developed countries such as the United States and France, the rate of infection peaks between the ages of 20 and 30 [4–8]. The prevalence of *H. pylori* in developing countries is between 85 and 95% while in developed countries, it ranged from 30 to 50%. Besides, after 2000, the prevalence of *H. pylori* infection was lower in European countries than before. However, this prevalence in Asian countries remained almost the same [9, 10]. *H. pylori* infection has different effects on human health, including both gastric and extra-gastric problems. Induced-gastric diseases include gastritis, peptic ulcer disease, functional dyspepsia, reflux disease, and gastric cancer. Extra-gastric complications of *H. pylori* infection include cardiopulmonary diseases (coronary artery disease and asthma), hematologic diseases (iron deficiency anemia and immune thrombocytopenic purpura), neurologic diseases (ischemic stroke, Parkinson, Alzheimer, and migraines), dermatologic diseases (chronic spontaneous urticaria), and metabolic diseases (metabolic syndrome and insulin resistance) [11–13]. There is considerable evidence linking *H. pylori* infection to extra-gastric diseases, but this evidence is contradictory. A review study was performed to determine the association of *H. pylori* infection with extra-gastric or gastrointestinal outcomes such as neurological, cutaneous, blood, ocular, cardiovascular, metabolic, allergic, as well as hepatic and biliary diseases, and the results showed that *H. pylori* was the cause of a number of gastrointestinal diseases, including the peptic ulcer disease and gastric adenocarcinoma, which are the result of interactions between factors, bacterial virulence, host and environmental factors. The results of this study also showed that many extra-gastric manifestations such as Alzheimer's disease, Multiple sclerosis, Parkinson's disease, Guillain-Barré syndrome, dermatological diseases, Psoriasis, chronic urticaria, Alopecia areata, autoimmune bullous diseases, hematologic diseases, iron deficiency anemia were related with *H. pylori* infections, but most of the evidence is from epidemiological studies which have not examined confounders and interactions. Therefore, these associations can currently be expressed as a hypothesis because it has not yet reached the stage of causal relation [14–19]. Some other studies have shown that *H. pylori* infection is involved in the onset of consequences such as vitamin B12 deficiency, insulin resistance, metabolic syndrome, diabetes, and non-alcoholic fatty liver, but they have suggested to perform more studies with greater sample sizes to confirm these results. Study of *H. pylori* can reveal the clinical facts of humans and bacteria as well as can help to clarify the effect of bacteria on humans [20–23]. Metabolic syndrome is a complex disorder defined by a combination of risk factors that increase the risk of atherosclerotic cardiovascular disease and type II diabetes [11]. So far, different criteria collections with different numbers of parameters were developed to identify metabolic syndrome, such as National Cholesterol Education Program Adult Treatment Panel III (NCEP ATPIII), World Health Organization (WHO), European Group for the study of Insulin Resistance (EGIR), American Association of Clinical Endocrinology (AACE), International Diabetes Federation (IDF), and American Heart Association/National Heart, Lung, and Blood Institute (AHA / NHLBI). Among all risk factors defined in different score systems, insulin resistance or impaired glucose tolerance is one of the major components for the diagnosis of metabolic syndrome [24–27]. In addition, the results of various studies have shown that *H. pylori* infection leads to chronic inflammation and immune system responses in the stomach and gastrointestinal tract [28–30]. As a result, some inflammatory cytokines and adipokines, such as tumor necrosis factor α (TNF-α) and leptin are involved in this inflammation and immune responses [31–33]. Patients with *H. pylori* infection have low leptin and high TNF-α levels compared to other populations [34, 35]. According to previous studies, low levels of leptin and high levels of TNF-α lead to insulin resistance [34, 35]. Finally, insulin resistance and central obesity are the main causes of metabolic syndrome [36]. Therefore, the presence of *H. pylori* infection may affect the incidence of insulin resistance and metabolic syndrome. However, it should be noted that this association can occur in the opposite way. Thus, people with metabolic syndrome or insulin resistance, such as obese people, may develop long-term *H. pylori* infection. The results of a meta-analysis showed that obese people were 46% more likely to develop *H. pylori* infection than lean people. The results of another study showed that the prevalence of *H. pylori* infection in diabetics was 50% and the chance of developing *H. pylori* infection in people with diabetes was 27% higher than other people in the community [37, 38]. The role of *H. pylori* infection on the risk of metabolic syndrome and insulin resistance has been investigated in several studies, but the results of different studies are contradictory. In this instance, several studies have shown a positive association between *H. pylori* and insulin resistance as well as metabolic syndrome [39–42] while other studies have shown no significant association between *H. pylori* and these consequences [43, 44]. Investigating the association between *H. pylori* infection and health outcomes such as metabolic syndrome or insulin resistance can help clinicians and specialists in early prevention and treatment as well as can provide new conditions for clinical research. Thus, the aim of the present study was to determine the pooled effect of the association between *H. pylori* infection and the risk of metabolic syndrome and insulin resistance using a combination of results of cohorts as well as case–control studies.

## Material and methods

This systematic review and meta-analysis was based on the Preferred Reporting Items for Systematic Reviews and Meta-analyses (PRISMA) which is dedicated to the systematic review and meta-analysis of observational studies.

### Search terms and complex search syntax

All original articles published from January 1990 to January 2021 were searched in international databases including Medline (PubMed), Web of Sciences, Scopus, EMBASE, and CINHAL without language restrictions. The search strategy was performed based on three root keywords of *H. pylori*, insulin resistance, and metabolic syndrome and their mesh terms including Campylobacter pylori, Helicobacter, Helicobacter nemestrinae, Campylobacter pylori subsp. pylori, Campylobacter pyloridis, insulin sensitivity, metabolic syndrome X, dysmetabolic syndrome X, metabolic cardiovascular syndrome, and cardiometabolic syndrome. Gray literature was then used to access unpublished articles, dissertations, and international reports. In addition, after the final selection of articles, a manual search was performed by reviewing the references of related articles. The search strategy in international databases was independently conducted by two researchers (MA and AM) and the disputes were resolved by a third person (YM).

### Eligibility criteria

In this research, a meta-analysis of studies aimed at determining the association of *H. pylori* infection with the occurrence of insulin resistance or metabolic syndrome was considered; meaning that exposure in these studies was the infection of *H. pylori* and the main outcome was the occurrence of insulin resistance or metabolic syndrome. Therefore, case–control and cohort studies were included in this meta-analysis. The statistical population studied in these initial studies was all individuals, whether they had a specific disease or they were healthy. There were no specific limitations in this research for the method of diagnosing *H. pylori* infection for inclusion of studies and it was decided that the results would be analyzed based on the diagnostic method of *H. pylori*. The definition of metabolic syndrome in this study was also based on its international definition, i.e. people who have risk factors such as dyslipidemia (elevated triglycerides and apolipoprotein B -containing lipoproteins, and low high-density lipoproteins (HDL)), elevation of arterial blood pressure (BP) and dysregulated glucose homeostasis, while abdominal obesity and/or insulin resistance (IR) [45, 46]. In addition, metabolic syndrome was considered according to the National Cholesterol Education Program—Adult Treatment Panel III (NCEP-ATP III) and the International Diabetes Federation (IDF) criteria [24, 47]. Homeostatic model assessment-insulin resistance (HOMA-IR) or similar criteria were used in the studies to determine insulin resistance.

Other studies including cross-sectional studies, case reports, case series, systematic reviews, meta-analyses, letters, and editorials were excluded.

### Data extraction

To extract information, first, a checklist including the first author's name, date of publication, country, type of study, sample size, type of diabetes, bacterial detection method, age (mean and its dispersion), gender (number of male), and effect size (odds ratio or risk ratio) was designed. Then, two authors (MA and AM) independently extracted the data based on the checklist, and the conflicts were resolved by a third person (YM).

### Quality assessment

In this study, to evaluate the quality of included articles, the Joanna Briggs Institute (JBI) critical appraisal checklist was used for case–control studies and cohorts. The purpose of these appraisals is to assess the methodological quality of a study and to determine the extent to which a study has addressed the possibility of bias in its design, conduct, and analysis. JBI critical appraisal tools have been developed by the JBI and collaborators and approved by the JBI Scientific Committee following extensive peer review. The checklist for case–control studies consisted of ten questions and that for the cohort studies were composed of eleven questions related to their methodology, which have been defined in the categories of ‘Yes’, ‘No’, ‘Not Applicable’, and ‘Unclear’. Finally, these studies were scored based on the number of Yes cases [48, 49].

### Statistical analysis

The effect sizes in this meta-analysis included the odds ratio in case–control studies and the risk ratio in cohort studies. To perform a meta-analysis, first, the logarithm and logarithm error of each of these indicators were estimated, then the results were combined using the random effect model and the pooled estimate of each of these indicators was calculated with a 95% confidence interval (95%CI). Egger’s test and funnel plot asymmetry were used to examine and determine the potential publication bias. In this study, the Cochran Q test and I^2^ statistics were used to assess statistical heterogeneity between studies. According to the Cochrane criteria and I^2^ index, the rate of heterogeneity was divided into 4 categories: 0% to 40% (may not be important), 30% to 60% (may represent moderate heterogeneity), 50% to 90% (may represent substantial heterogeneity), and finally 75% and above (considerable heterogeneity) [50–53]. Sensitivity analysis was performed to determine the overall effect without considering any of the initial studies. All statistical analyses were performed using STATA 16.0 (Stata Corp., College Station, TX, USA).

## Results

### Study characteristics

**Fig. 1:**
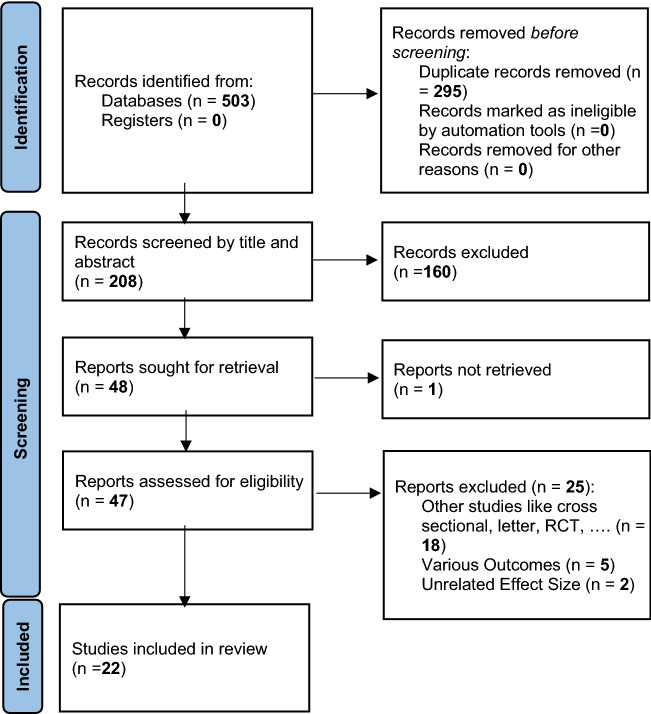
PRISMA 2020 flow diagram for new systematic reviews which included searches of databases and registers only

After completing the search in international databases, 782 studies were retrieved. After removal of duplicates, screening by titles, abstracts, and full texts was performed considering inclusion and exclusion criteria; 22 studies remained to meta-analyze the association between *H. pylori* infection and insulin resistance and metabolic syndrome (Fig. 1). The characteristics of the studies included in this meta-analysis were reported in Table 1.

**Table 1:** The characteristics of studies evaluating the effect of *H. pylori* infection on the risk of metabolic syndrome and insulin resistance in different populations

Authors (Years) (R)	Country	Type of Study (Cohort or case control)	Study Population (Children, Adults, Women, or ……)	Type of diabetes (IDDM, NIDDM, GDM)	*H. pylori* detection methods	Outcome (metabolic syndrome or insulin resistance)	Age (Mean)	BMI (Mean)	Effect size (OR with a 95% CI) or (RR with a 95% CI)	
Gunji et al. (2008) [54]	Japan	Case control	9582 Japanese (5488 men and 1906 women)		IgG antibody without further laboratory assessment	Metabolic syndrome (Japanese criteria)	47.3	22.9	1.39 (1.18–1.62)	
So et al. (2009) [55]	China	Case control	288 men (107 Low adiponectin and 181 Normal adiponectin)		IgG antibody concentrations were measured by a two-step immunometric assay (Immulite)	Insulin resistance	40.7	25.3	1.30 (1.02, 1.65)	
Jeon et al. (2012) [40]	USA	Cohort	63	Diabetes	Type-specific IgG anti-body responses	Insulin resistance	66.8	30.1	(HR) Sex and education adjusted	(HR) Multivariate analysis
									2.42 (0.99–5.92)	2.6 (1.10–6.60)
Naja et al. (2012) [56]	Lebanon	Case control	308 Lebanese adults (*H. pylori* (+) 160 And *H. pylori* (−)148)		Immunoglobulin G antibody titers	Metabolic syndrome (IDF)	40.97	27.3	Bivariate logistic regression: 0.71 (0.44–1.12)	
									Multivariate logistic regression: 0.54 (0.19–1.51)	
Naja et al. (2012)[56]	Lebanon	Case control	308 Lebanese adults (*H. pylori* (+) 160 And *H. pylori* (−)148)		Immunoglobulin G antibody titers	Insulin resistance	40.97	27.03	Bivariate logistic regression: 0.70 (0.43–1.16)	
									Multivariate logistic regression: 0.74 (0.40–1.36)	
Shin et al. (2012) [71]	South Korea	Cohort	5889 included subjects (3297 men and 2592 women)		Anti-HP immunoglobulin G (IgG) antibody titers + detection of HP by histologic analysis	Metabolic syndrome (NCEP)	48.0	23.8	serological status	histologic status
									IDF: 1.29 (1.10–1.50)	1.29 (1.11–1.50)
									NCEP: 1.30 (1.13–1.50)	1.31 (1.15–1.50)
Hsieh et al. (2013) [72]	Taiwan	Cohort	2070 participants (*H. pylori* (+) 903 And *H. pylori* (−)1167)	NIDDM	RUT	Insulin resistance	57.16	23.5	1.61	
Bajaj et al. (2014) [57]	India	Case control	140 (aged ≥ 18 years) participants (80 type 2, 60 controls)	NIDDM	Rapid urease tests, histological examination of antral endoscopic biopsy specimens and serology	Insulin resistance	55.6	–	2.4	
Malamug et al. (2014)[58]	USA	Case control	4,136 aged 18 and over (NHW 1949, NHB 853, MA 1334)		IgG anti- bodies to *H. pylori* in human serum	Insulin resistance	50	29.40	Male	Female
									NHW 1.6 (1.2–2.2)	1.8 (1.31–2.54)
									NHB:1.5 (1.03–2.37)	1.3 (0.92–1.98)
									MA :1.4 (0.99–2.05)	1.8 (1.32–2.66)
Vafaeimanesh et al. (2014)[59]	Iran	Case control	429 (211 diabetic, 218 without diabetes)	NIDDM	Anti-HP IgG anti- body	Insulin resistance	51	–	1.26	
Chen et al. (2015) [60]	Taiwan	Case control	811 Residents Younger than 50 Years Old (*H. pylori* (+)509 And *H. pylori* (−)302)		*H. pylori*-specific immunoglobulin G (IgG) antibody	Metabolic syndrome (NCEP)	59.2	24.9	3.717(1.086–12.719)	
Chen et al. (2015) [61]	Taiwan	Case control	3578 subjects ((*H. pylori* (+)724 And *H. pylori* (−)2854)		UBT	Metabolic syndrome (NCEP)	39.8	24.9	Male: 1.76 (1.26–2.47)	
									Female: 3.11 (1.73–5.62)	
Sayilar et al. (2015) [73]	Turkey	Cohort	200 patients (*H. pylori* (+)99 And *H. pylori* (−)101)		*H. pylori* was observed in the biopsy specimens by microscopic examination of the slides stained with hematoxylin and eosin	Metabolic syndrome (NCEP)	48.1	30.1	3.61 (2.46–5.30)	
Kayar et al. (2015) [42]	Turkey	Case control	133 dyspeptic patients (*H. pylori* (+)71 And *H. pylori* (−)62)		*H. pylori* antigen stool test	Insulin resistance	–	–	1.47	
Chen et al. (2016) [62]	Taiwan	Case control	2113 MS + 557, MS− 1556)		C-UBT	Metabolic syndrome (NCEP)	59.9	27.3	1.50 (1.20–1.87)	
Takeoka et al. (2016) [63]	Japan	Case control	1044 participants (*H. pylori* (+)247 And *H. pylori* (−)797)		HP-specific IgG measured	Metabolic syndrome (NCEP)	46.6	22.4	IgG concentration	
									Moderate: 3.47 (0.83–23.9)	High: 3.70 (0.62–71.3)
Alzahrani et al. (2017) [64]	USA	Case control	842(421 adults with newly diagnosed diabetes and 421 matched controls)	NIDDM	*H. pylori* immunoglobulin G (IgG) antibody in serum	Insulin resistance	49.6	35.6	1.03(0.74–1.42)	
Allam et al. (2018) [65]	Egypt	Case control	80 patients (*H. pylori* (+)40 And *H. pylori* (−)40)		microscopy of histological sections stained with Giemsa stain	Insulin resistance	30.4	24	0.642 (0.525–0.767)	
Alshareef et al. (2018) [66]	Sudan	Case control	166 women (20 GDM + , 146 GDM−)	GDM	Helicobacter pylori IgG antibodies	Insulin resistance	26.5	26.2	2.8 (1.1–7.5)	
Refaeli et al. (2018) [67]		Case control	147,936 individuals 25–95 years		UBT	Metabolic syndrome (IDF)	42.8	–	1.15 (1.10–1.19)	
Chen et al. (2019) [68]	Taiwan	Case control	6024 adults		RUT	Metabolic syndrome (THPA)	51.6	25.19	1.26 (1.00–1.57)	
Chen et al. (2019) [68]	Taiwan	Case control	6024 adults	DM	RUT	Insulin resistance	51.6	25.19	1.59 (1.17–2.17)	
Lim et al. (2019) [69]	South Korea	Case control	15,195 subjects (*H. pylori* (+)6569 And *H. pylori* (−)8626)		Serum HP immunoglobulin G antibody (anti-HP IgG)	Metabolic syndrome (NCEP)	50.7	23.5	1.19 (1.09–1.31)	
Yu et al. (2019) [70]	China	Case Control	5884 participants		C-UBT for the detection of *H. pylori*	Metabolic syndrome (IDF)	50.9	26.8	1.21 (1.02–1.36)	

OR: odds ratio; RR: risk ratio; HP: *Helicobacter pylori*; NIDDM: Non-insulin-dependent diabetes mellitus; RUT: rapid urease test; UBT: urea breath test; GDM: gestational diabetes mellitus; NCEP: National Cholesterol Education Program; IDF: International Diabetes Federation; IDDM: Insulin-dependent diabetes mellitus; THPA: The Taiwan Health Promotion Administration of the Ministry of Health and Welfare; CI: confidence interval; BMI: body mass index

For this meta-analysis, 18 case–control studies [42, 54–70] were included, of which 9 studies [54, 56, 60–63, 67, 69, 70] determined the association of *H. pylori* infection with the occurrence of metabolic syndrome and 9 studies [42, 55, 57, 58, 64–66, 68] determined the association of *H. pylori* infection with the occurrence of insulin resistance in patients with diabetes. Besides, 4 cohort studies [40, 71–73] were analyzed, of which 2 studies [71, 73] examined the presence of *H. pylori* infection in connection with metabolic syndrome and 2 studies [40, 72] on insulin resistance (Table 1).

### Association of *H. pylori* infection with metabolic syndrome (a combination of case–control studies)

**Fig. 2:**
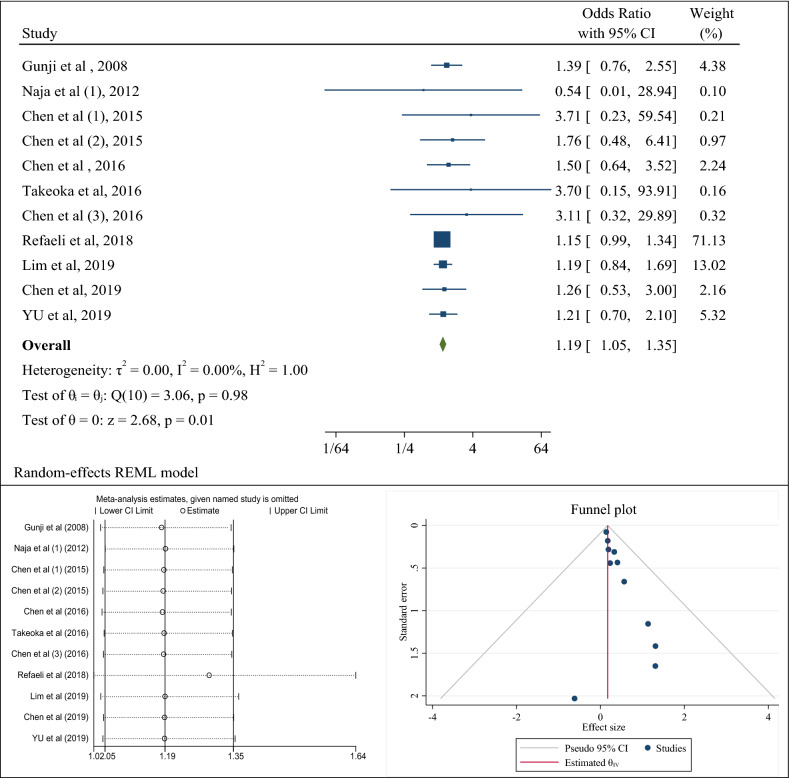
The odds ratio (OR) between *Helicobacter pylori* infection and the occurrence of metabolic syndrome, sensitivity analysis and publication bias using a combination of the results of case–control studies (CI: Confidence Interval)

9 case–control studies were evaluated the association of *H. pylori* infection with the occurrence of metabolic syndrome. After combining the results of these case–control studies, the pooled estimate of odds ratio was 1.19 (95% CI 1.05–1.35). The rate of heterogeneity was significantly lower and equal to zero percent with a significant level of 0.98 (Fig. 2a). The results of publication bias analysis showed that although in the funnel plot, there was a heterogeneity between studies, the results of the eggers test showed the absence of publication bias (B = 0.54; SE = 0.40; P-value = 0.181) (Fig. 2b). Besides, sensitivity analysis showed that if each study was omitted, the pooled odds ratio and its 95% confidence interval would be in line with the overall estimates, except for the study by Refeali et al. [67], which, if omitted, increased the pooled odds ratio to 1.29 (95% CI 1.02–1.64) (Fig. 2c).

In Table 2, subgroup analysis was performed based on the diagnostic method of *H. pylori*. The results showed that regarding the diagnostic method of anti- *H. pylori* antibody, the pooled odds ratio was 1.26 (95% CI 1.01–1.70) and according to the C urea breath test (UBT), it was 1.17 (95% CI 1.02–1.35; *p* value: 0.910). In this meta-analysis, the results of subgroup analysis based on the variable of body mass index showed that people with *H. pylori* infection, who had a body mass index above 24, had a higher chance of developing metabolic syndrome than people with a body mass index below 24 (OR: 1.36; 95% CI 1.01–2.00 than OR: 1.25; 95% CI 0.92–1.69). Also, people with an infection, who were older than 45 years, had a higher chance of developing metabolic syndrome than other people (OR: 1.26; 95% CI 1.00–1.89 than OR: 1.16; 95% CI 1.00–1.35) (Table 2). The results of meta-regression analysis also showed that with increasing body mass index and age, the chances of developing metabolic syndrome in people with *H. pylori* infection increased although the results of meta-regression were not statistically significant (P: 0.885) (Fig. 5).

**Table 2:** Determining the odds ratio with the confidence interval of association between Helicobacter pylori infection and metabolic syndrome and insulin resistance in case–control studies based on variables of detection methods of infection, study populations, age, body mass index, and the continents of the world

Subgroup		Number of studies	Summery odds ratio (95% CI)	Between studies			Between subgroups	
				I^2^	P _heterogeneity_	Q	Q	P _heterogeneity_
Metabolic Syndrome	Method of bacteria detection							
	Anti-* H. pylori* antibody	5	1.26 (1.01–1.70)	0.0%	0.852	1.38	0.19	0.910
	Rapid urease test	1	1.26 (0.53–3.00)	–	–	–		
	C urea breath test (UBT)	5	1.17 (0.02–1.35)	0.0%	0.830	1.49		
	BMI						0.13	0.71
	< = 24	3	1.25 (0.92–1.69)	0.0%	0.731	0.62		
	> 24	5	1.36 (1.01–2.00)	0.0%	0.977	1.63		
	Age							0.56
	< = 45	5	1.16 (1.00–1.35)	0.0%	0.771	1.78	0.32	0.32
	> 45	6	1.26 (1.00–1.89)	0.0%	0.964	0.96		
Insulin Resistance	Method of bacteria detection							
	Anti-* H. pylori* antibody	7	1.63 (1.25–2.12)	0.0%	0.990	2.71	0.44	0.661
	Rapid urease test (RUT) & Histology	3	1.33 (0.56–3.16)	67.1%	0.043	6.64		
	Type of diabetes							
	Diabetes dellitus	5	1.19 (1.00–1.78)	0.0%	0.890	4.37	3.33	0.192
	Gestational diabetes	1	2.80 (0.39–19.92)	–	–	–		
	NIDDM	4	1.80 (1.34–2.42)	0.0%	0.510	2.33		
	Population							
	Male	4	1.19 (1.00–1.78)	0.0%	0.990	0.08	0.23	0.89
	Female	4	2.80 (0.39–19.92)	0.0%	0.940	0.38		
	Both	7	1.80 (1.34–2.42)	44.1%	0.152	9.36		
	Continent							
	Asia	5	1.67 (1.23–2.26)	0.0%	0.764	1.85	1.72	0.63
	America	7	1.47 (0.89–2.44)	0.0%	0.930	0.58		
	Africa	2	1.00 (0.26–3.77)	47.6%	0.173	1.91		
	Europe	1	2.35 (1.16–4.73)	–	–	–		
	BMI							
	< = 29	5	1.04 (0.60–1.79)	12.2%	0.488	3.46	0.84	0.36
	> 29	7	1.47 (0.89–2.44)	0.0%	0.662	0.55		
	Age							
	<= 45	8	1.09 (0.70–1.70)	2.5%	0.770	4.05	0.99	0.77
	> 45	7	1.77 (1.35–2.33)	0.0%	0.872	2.40		

OR: odds Ratio, I^2^: I Square, Q: Q Cochrane Test, CI: confidence interval, BMI: body mass index

### Association of *H. pylori* infection with insulin resistance (a combination of case–control studies)

**Fig. 3:**
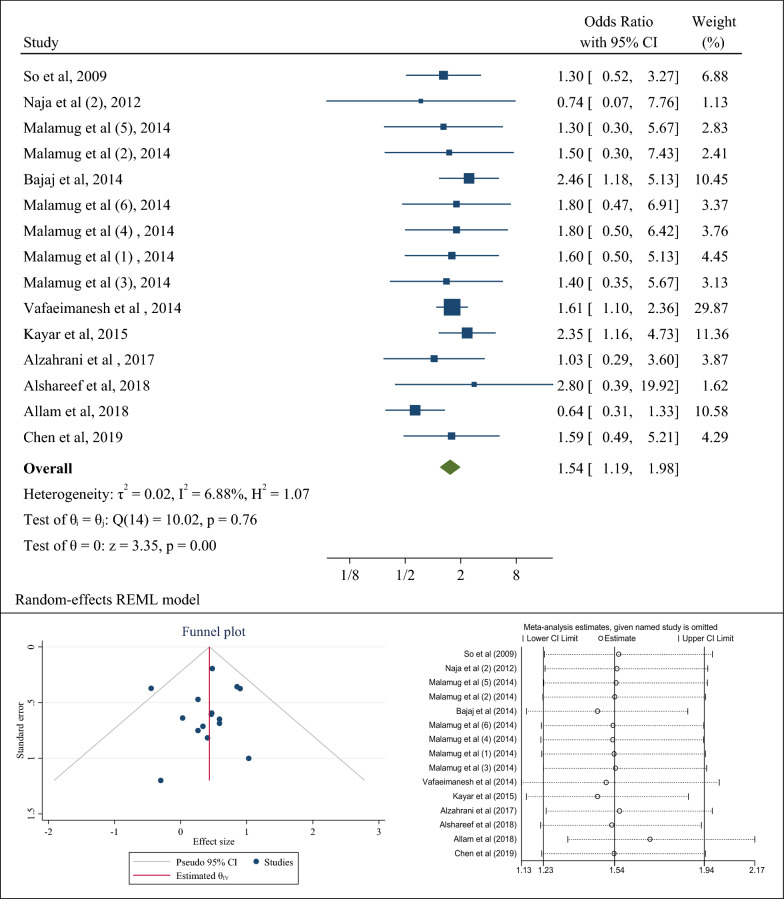
The odds ratio (OR) between *Helicobacter pylori* infection and insulin resistance, sensitivity analysis and publication bias using a combination of the results of case–control studies (CI: Confidence Interval)

10 case–control studies evaluated the association between *H. pylori* infection and the occurrence of insulin resistance in patients with diabetes. After combining the results of these case–control studies, the pooled odds ratio was 1.54 (95% CI 1.19–1.98) (Fig. 3a).

The results of the Eggers test and funnel plot showed no publication bias (B = − 0.17; SE = 0.61; P-value = 0.791) (Fig. 3b). In addition, the sensitivity analysis showed that if any of the studies were omitted, the pooled odds ratio would be in line with the overall pooled odds ratio result. Only if the study of Allam et al. [65] were removed, the pooled odds ratio would be equal to 1.70 (95% CI 1.33–2.16) (Fig. 3c).

In Table 2, subgroup analysis was performed based on the diagnostic method of *H. pylori*, types of diabetes, gender of population, and continent. The results showed that regarding the diagnostic method of anti-*H. pylori* antibody, the pooled odds ratio for the occurrence of insulin resistance in patients with diabetes with *H. pylori* infection was equal to 1.63 (95% CI 1.25–2.12) and according to the diagnostic method of the rapid urease test (RUT) & histology, it was equal to 1.33 (95% CI 0.56–3.16; *p* value: 0.661). In the subgroup analysis, 5 studies of the population with diabetes (without specifying the type of diabetes) were examined, which has been named in this analysis as the category of diabetes mellitus. The results showed that the occurrence chance of insulin resistance in diabetes mellitus patients with *H. pylori* infection was 1.19 (95% CI 1.00–1.78), and in type II patients with diabetes with *H. pylori* infection, it was 1.80 (95% CI 1.34–2.42; *p* value: 0.192). The subgroup analysis based on the continent of study showed the odds ratio of the presence of *H. pylori* infection and the occurrence of insulin resistance in Asian population was 1.67 (95% CI 1.23–2.26), and the corresponding estimates in America and Africa were 1.47 (95% CI 0.89–2.44) and 1.00 (95% CI 0.26–3.77; *p* value: 0.63), respectively. The results of subgroup analysis to determine the association between *H. pylori* infection and the occurrence of insulin resistance in diabetic patients based on body mass index and age in Table 2 showed that diabetics with *H. pylori* infection and body mass index higher than 29 were more likely to develop insulin resistance, compared to diabetic people with an infection and a body mass index lower than 29 (OR: 1.47; 95% CI 0.89–2.44 than OR: 1.04; 95% CI 0.60–1.79) albeit it was not statistically significant. Also, diabetics with *H. pylori* infection over the age of 45 years were more likely to develop insulin resistance than diabetics with the infection under the age of 45 years (OR: 1.77; 95% CI 1.35–2.33 than OR: 1.09; 95% CI 0.70–1.70) (Table 2). The results of meta-regression analysis also showed that with age, the chances of developing insulin resistance in diabetics with *H. pylori* infection increased although the results of meta-regression were not statistically significant (P: 0.993) (Fig. 5).

### Association of *H. pylori* infection with insulin resistance and metabolic syndrome (a combination of cohort studies)

**Fig. 4:**
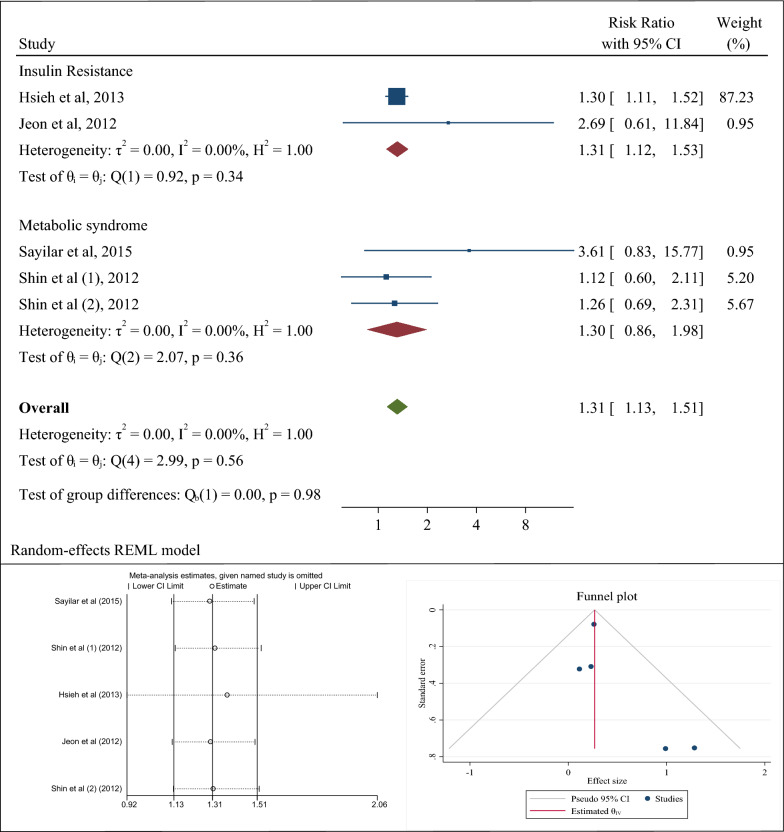
The risk ratio (RR) between *Helicobacter pylori* infection and metabolic syndrome and insulin resistance, sensitivity analysis and publication bias using a combination of the results of cohort studies (CI: Confidence Interval)

**Fig. 5:**
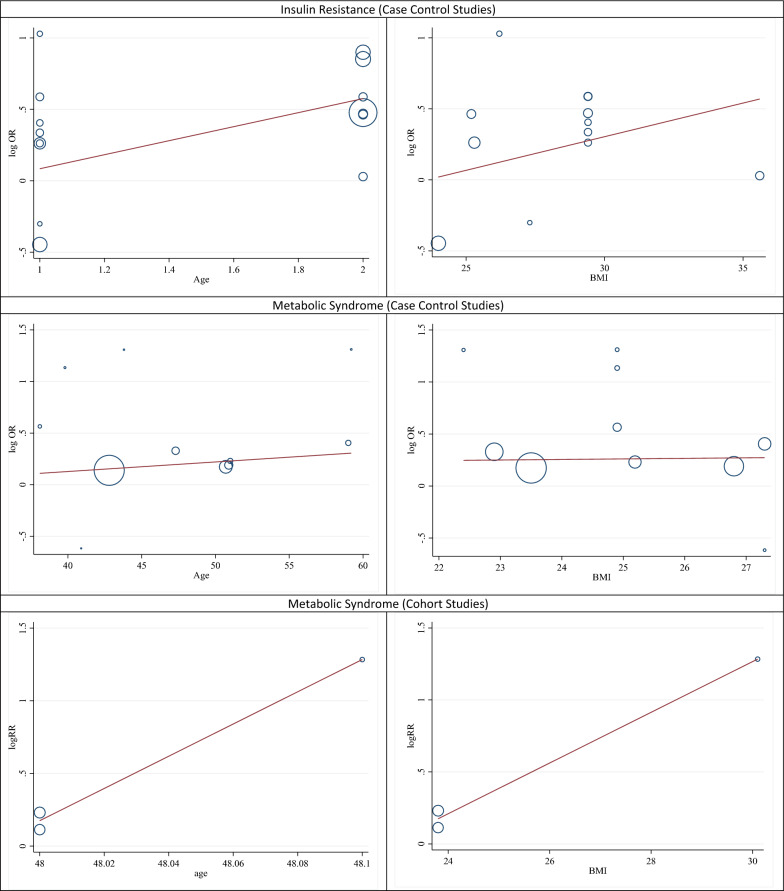
The meta-regression results in the effect of age and BMI (Body Mass Index) on the association between *H. pylori* and insulin resistance or metabolic syndrome

Finally, cohort studies were evaluated. Two cohort studies determined the association between *H. pylori* infection and the occurrence of insulin resistance in patients with diabetes; in the study by Hsieh et al. [72], the risk ratio of *H. pylori* infection and insulin resistance was 1.30 (95% CI 1.11–1.52) and in the other study by Jeon et al. [40], the corresponding risk ratio was 2.69 (95% CI 0.61–11.84). Also, two cohort studies evaluated the association of *H. pylori* with metabolic syndrome in general population; in the study by Sayilar et al. [73], the odds ratio was 3.61 (95% CI 0.83–15.77) and in the other study by Shin et al. [71], the risk ratio based on histological diagnosis for *H. pylori* was 1.26 (95% CI 0.69–2.31) and the risk ratio based on serological diagnosis for *H. pylori* was 1.12 (95% CI 0.60–2.11). In the analysis of cohort studies, it is better to consider the final result which is equal to the risk ratio of 1.31 or the confidence interval of 1.13 to 1.51. As a result, the risk of developing metabolic syndrome or insulin resistance in a population with *H. pylori* infection (whether diabetic or healthy) is 31% higher than that in a population free of *H. pylori*. The heterogeneity rate was zero percent (Fig. 4a–c). In this section, the number of studies was low and therefore it was not possible to perform subgroup analyzes to examine the association, but meta-regression results to investigate the role of age and body mass index of the patients with *H. pylori* infection in the incidence of metabolic syndrome showed that with age and body mass index, the incidence of metabolic syndrome increased in patients with infection, but it was not statistically significant (P: 0.559) (Fig. 5).

### Quality assessment

In the present meta-analysis, the quality of 18 case–control studies and 4 cohort studies was evaluated using JBI critical appraisal tools and the results of which were presented in Tables 3 and 4, respectively. The quality assessment checklist of the case–control studies showed that most of these studies had a high-quality score. Except for the study of Chen et al.[68], which received a score of 6, the rest of the studies had a high-quality score of more than 6 (Table 3). The quality assessment of cohort studies showed high scores for quality of all studies (Table 4).

**Table 3:** Quality assessment of case–control studies based on the JBI critical appraisal checklist

Studies	Q1	Q2	Q3	Q4	Q5	Q6	Q7	Q8	Q9	Q10	Total Score
Gunji et al. (2008) [54]	Y	Y	N	N	Y	Y	Y	N	Y	Y	7
So et al. (2009) [55]	Y	Y	Y	Y	Y	Y	Y	N	Y	Y	9
Naja et al. (2012) [56]	N	Y	N	Y	Y	Y	Y	Y	Y	Y	8
Bajaj et al. (2014) [57]	Y	Y	Y	Y	Y	Y	N	N	Y	Y	8
Malamug et al. (2014) [58]	Y	Y	Y	N	Y	Y	Y	Y	Y	Y	9
Vafaeimanesh et al. (2014) [59]	N	Y	N	Y	Y	Y	Y	Y	Y	Y	8
Chen et al. (2015) [60]	N	Y	Y	Y	Y	Y	Y	N	Y	Y	8
Chen et al. (2015) [61]	N	Y	Y	N	Y	Y	Y	Y	Y	Y	8
Kayar et al. (2015) [42]	Y	Y	N	Y	Y	N	N	Y	Y	Y	7
Chen et al. (2016) [62]	N	Y	Y	Y	Y	Y	Y	N	Y	Y	8
Takeoka et al. (2016) [63]	Y	Y	N	Y	Y	Y	Y	Y	Y	Y	9
Alzahrani et al. (2017) [64]	Y	Y	N	Y	Y	Y	Y	Y	Y	Y	9
Allam et al. (2018) [65]	N	Y	Y	Y	Y	Y	Y	Y	Y	Y	9
Alshareef et al. (2018) [66]	Y	Y	N	Y	Y	Y	Y	N	Y	Y	8
Refaeli et al. (2018) [67]	N	Y	N	N	Y	Y	Y	Y	Y	Y	7
Chen et al. (2019) [68]	N	Y	N	N	Y	Y	Y	N	Y	Y	6
Lim et al. (2019) [69]	N	Y	N	Y	Y	Y	Y	N	Y	Y	7
Yu et al. (2019) [70]	N	Y	N	N	Y	Y	Y	Y	Y	Y	7
Q1: Were the groups comparable other than the presence of disease in cases or the absence of the disease in controls?											
Q2: Were cases and controls matched appropriately?											
Q3: Were the same criteria used for identification of cases and controls?											
Q4: Was exposure measured in a standard, valid and reliable way?											
Q5: Was exposure measured in the same way for cases and controls?											
Q6: We’re confounding factors identified?											
Q7: Were strategies to deal with confounding factors stated?											
Q8: Were outcomes assessed in a standard, valid and reliable way for cases and controls?											
Q9: Was the exposure period of interest long enough to be meaningful?											
Q10: Was appropriate statistical analysis used?											

Y: Yes; N: No; UC: Unclear; NP: Not applicable

**Table 4:** Quality assessment of cohort studies based on the JBI critical appraisal checklist

Studies	Q1	Q2	Q3	Q4	Q5	Q6	Q7	Q8	Q9	Q10	Q11	Total Score
Jeon et al (2012) [40]	Y	Y	Y	Y	Y	Y	Y	N	Y	Y	Y	10
Shin et al (2012) [71]	Y	Y	Y	Y	Y	Y	Y	Y	UC	UC	Y	9
Hsieh et al (2013) [72]	Y	Y	Y	Y	Y	Y	Y	N	Y	UC	Y	9
Sayilar et al (2015) [73]	Y	Y	Y	Y	Y	Y	Y	Y	N	N	Y	9
Q1: Were the two groups similar and recruited from the same population?												
Q2: Were the exposures measured similarly to assign people to both exposed and unexposed groups?												
Q3: Was the exposure measured in a valid and reliable way?												
Q4: Were confounding factors identified?												
Q5: Were strategies to deal with confounding factors stated?												
Q6: Were the groups/participants free of the outcome at the start of the study (or at the moment of exposure)?												
Q7: Were the outcomes measured in a valid and reliable way?												
Q8: Was the follow up time reported and sufficient to be long enough for outcomes to occur?												
Q9: Was follow up complete, and if not, were the reasons to loss to follow up described and explored?												
Q10: Were strategies to address incomplete follow up utilized?												
Q11: Was appropriate statistical analysis used?												

Y: Yes; N: No; UC: Unclear; NP: Not applicable

## Discussion

This meta-analysis aimed to determine the association between *H. pylori* infection and the occurrence of metabolic syndrome and insulin resistance. The results showed that the presence of *H. pylori* infection was associated with the risk of metabolic syndrome and insulin resistance in society. These results were in the same line with other studies conducted in the world. The results of previous studies have shown that one of the extra-gastric complications of *H. pylori* infection was the occurrence of insulin resistance in patients [59, 74–76]. In this instance, various pathophysiologic pathways have been suggested that induce insulin resistance by *H. pylori*, such as activation of pro-inflammatory substances (CRP, PAI-1, and TNF-α), production of reactive oxygen species (ROS), alteration of ghrelin and leptin levels, and increased production of lipopolysaccharides [40, 77–79]. On the other hand, the virulent strains of *H. pylori* (cag + with induction of inflammatory factors 71, IL6, CRP) and chronic inflammation affect the insulin-regulating gastroduodenal hormones and ultimately predispose the person to insulin resistance [40, 80]. Inflammation caused by *H. pylori* affects insulin-producing pancreatic B cells and reduces insulin secretion [76, 81]. By acting on the hormone somatostatin, cag + strains reduce insulin secretion by the pancreas. In addition, *H. pylori* increase the levels of leptin and ghrelin and predispose people to obesity and diabetes [76, 82]. *H. pylori* cause type II diabetes by disrupting the production and release of plasma lipoproteins and disrupting glucose tolerance and Hb1Ac levels. On the other hand, patients with diabetes are also exposed to *H. pylori* because the humoral and cellular immune systems have been damaged and the person is susceptible to the bacterium. Decreased gastric motility in patients with diabetes reduces gastric acid and provides a basis for colonization of *H. pylori* strains [83, 84]. Finally, it is concluded that cag + strains of *H. pylori* can predispose people to type II diabetes. Therefore, people with diabetes are always at risk for *H. pylori* [83–88]. Also, lipopolysaccharides from gram-negative bacteria such as *H. pylori*, may activate Toll-like receptors and subsequently develop insulin resistance [89]. Finally, all of these factors are among the reasons which can be attributed to the effect of *H. pylori* infection on the occurrence of insulin resistance and metabolic syndrome. The results of this meta-analysis on determining the association between *H. pylori* infection and the occurrence of metabolic syndrome were consistent with the meta-analysis of Upala et al. [90] in which, the results showed that people with *H. pylori* infection were 1.34 times more likely to develop metabolic syndrome. However, this study was conducted in 2016 and a total of 6 studies had been included in the meta-analysis [90]. Many studies have shown that different eating habits play a role in the development of *H. pylori* infection, and consequently the occurrence of metabolic syndrome is not unexpected. Consumption of fruits and vegetables is common in different cultures and in these communities, the risk of *H. pylori* infection is low due to the presence of antioxidants, especially vitamin C [91, 92]. On the other hand, consumption of some food items such as garlic or green pepper has been shown to be inversely related to the incidence of *H. pylori* infection. Excessive salt intake, or a high-salt diet, or high sugar intake, such as high sugar intake with black tea, are factors which may increase the incidence of *H. pylori* infection [93, 94]. Infection with *H. pylori* leads to lower levels of ghrelin and leptin compared to those in other healthy people of the community, which in turn increases obesity and metabolic syndrome [34, 35].

This study had some limitations and strengths. This meta-analysis was an updated study that considered the meta-analysis of Upala et al. [90] conducted in 2016. Upala et al. [90] had performed a meta-analysis with six studies, including case–control, cohort, and cross-sectional studies, and finally, they had reported the pooled effect size. One of the main limitations of the study by Upala et al. [90] was that they did not have a precise search strategy to obtain the initial studies. On the other hand, in epidemiology and methodology, it is not fundamentally correct to combine cross-sectional studies or the effect size obtained from these studies with cohort and case–control studies, and finally to report pooled effect size. Also, the effect size of cohort and case–control studies can be combined only if the prevalence of the desired outcome in the study population is less than 0.05, which has not been observed in the study of Upala et al. [90]. The present meta-analysis was performed with 22 case–control and cohort studies.

In the results of subgroup analysis, the association between the presence of *H. pylori* infection and the incidence of metabolic syndrome was different based on body mass index above and below 24. People with *H. pylori* infection and a high body mass index were more likely to develop metabolic syndrome. This association can be examined from several aspects. First, it is possible that the presence of *H. pylori* infection is associated with the incidence of high body mass index and ultimately obesity or overweight, which has been confirmed in previous studies, especially in the study of Baradaran et al. in 2021. On the other hand, obesity may exacerbate the association between infection and metabolic syndrome and play an interaction role. Additionally, age can also play a role as an interaction variable with obesity in increasing the association between *H. pylori* infection and metabolic syndrome. Infection with *H. pylori* leads to lower levels of ghrelin and leptin compared to other healthy people in the community, which in turn increases obesity and metabolic syndrome. Low levels of ghrelin lead to a delay in satiety when eating, which leads to increased overeating and ultimately obesity [37, 38]. Virulent *H. pylori* strains induce insulin-regulating gastroduodenal hormones by inducing inflammatory factors IL6, CRP, and chronic inflammation, and ultimately increase insulin resistance. Inflammation caused by *Helicobacter pylori* also affects insulin-producing pancreatic B cells and reduces insulin secretion. Cag + strains reduce insulin secretion by the pancreas by acting on the gastric hormone somatostatin. *H. pylori* causes type II diabetes by impairing the production, releasing plasma lipoproteins and disrupting glucose tolerance and Hb1Ac levels [37, 38].

One of the limitations of our study was the lack of a history of drug or non-drug treatment in patients with *H. pylori* infection. It plays a role in the presence of *H. pylori* infection and the incidence of metabolic syndrome and insulin resistance. Another limitation of this study was the lack of subgroup analysis based on different methods for identifying and diagnosing metabolic syndrome and insulin resistance because the initial studies have used different criteria to identify these two outcomes. On the other hand, the use of different criteria to identify the consequences of this meta-analysis can be considered as a source of creating heterogeneity between the initial studies during the meta-analysis. One of the limitations of this study was the lack of analysis and subgroup analyzes based on a series of confounding variables such as receiving treatment and the type of medication, due to the lack of reporting these variables in the initial studies. This limitation may affect the interpretation of the study results. On the other hand, cohort studies are the most important ones to investigate the association between clinical trial studies and are of great importance among observational studies, but in this meta-analysis, their number was low so that the study of the association was difficult. In the analysis of cohort studies, only two studies examined the association between *H. pylori* infection and the occurrence of insulin resistance in diabetic patients, and one of them has taken on very much weight. This may affect the main result and therefore more cohort studies, which are one of the strongest studies in determining the causal relation, are needed in this regard to examine this association.

## Conclusion

In this meta-analysis, the results showed that there was a possibility of metabolic syndrome and insulin resistance in case of *H. pylori* infection, but this association needed further investigation in studies, especially cohort ones with high sample sizes. This association can be considered as a warning at the moment but health policy makers should think about planning and implementing the interventions.

## Data Availability

Input data for the analyses are available from the corresponding author on request.

## Abbreviations

CI: Confidence interval
OR: Odds ratio
IDDM: Insulin-dependent diabetes mellitus
NIDDM: Non-insulin-dependent diabetes mellitus
CINAHL: Cumulative Index to Nursing and Allied Health Literature
EMBASE: Excerpta Medica dataBASE
STROBE: Strengthening the Reporting of Observationally Studies in Epidemiology
PRISMA: Preferred reporting items for systematic reviews and meta-analyses
*H. pylori*: *Helicobacter pylori*
